# 

**Published:** 1892-12

**Authors:** 


### CONTENTS.

ORIGINAL ARTICLES—Tracheotomy for Foreign Bodies in the Air Passages—By W. F. Westmoreland, M. D. .......... 631
The Early Diagnosis and Early Treatment of Acute Anterior Poliomyelitis—By A. Church, M. D. .......... 736
Air Pressure an Availabie Force in Retentive Dressing for Fractures of the Upper Dental Arcade—By J. R. Barret, M. D. .......... 643
Gonorrhœa in the Female—By R. R. Kime, M. D. .......... 648
CORRESPONDENCE—Our New York Letter .......... 652
SOCIETY NOTES—Southern Surgical and Gynecological Association .......... 657
EDITORIALS—Present Status of Thoracic Surgery .......... 674
Higher Medical Legislation .......... 677
Jefferson Medicai College .......... 678
BOOK REVIEWS .......... 663-6
Diseases of the Stomach; By C. A. Ewald, M. D.—The Physician's Visiting List for 1893.—The Medical News Visiting List for 1893.—The Physician's Pocket Day-Book.—Over One Thousand Prescriptions.
SELECTIONS AND ABSTRACTS .......... 666-673
The Deadly Policeman's Club and the Brutal Clubber.—The Relations of Pelvic Disease to Physical Disturbances in Women.—Ectopic Pregnancy.—Ulceration Endocarditis Consecutive to Gonorrhœa.—Study of Post-Diphtheritic Paralyses.—Gonorrhœal Infection of the Mouth in New-Born Infants.—The Corset as a Factor in Pelvic Diseases.—Periodic Nocturnal Cough in Infants.
PRESCRIPTION DEPARTMENT .......... 682–683
SPECIAL NOTES .......... 679-681

